# Attempted Self-Operation for Recurrent Appendicitis

**Published:** 1916-05

**Authors:** 


**Attempted Self-Operation for Recurrent Appendicitis.** F. P. Hulen, Pond Creek, Okla., Railway Surg. Jour., Feb. A man aged 19, married, who had had two previous attacks during which the advantages of radical operation was urged, and who was under medical treatment for the third, feeling himself worse, although the doctor (the author's son) had already been summoned, poured carbolic acid over the lower abdomen and exposed the viscera with a Barlow knife which he had used recently for castrating pigs. He fainted and fell off the bed onto the floor, the intestines coming in contact with soiled rugs. The doctor arrived almost immediately, washed off the intestines with 1:4000 bichlorid and sent for the author who completed the operation. Very little anaesthetic was needed on account of the local action of the carbolic acid. Recovery occurred by first intention but the skin necrosis due to the carbolic acid did not heal for six weeks as skin grafting was refused. In the discussion, W. H. German of Chicago narrated the case of a medical missionary in China who instructed his wife in the details of operation and she anaesthetized him and performed a successful appendectomy, although she was not even a trained nurse. (Note: While an interne in Rochester, we saw a case of self-castration which had been successfully and radically performed not for pathologic but physiologic manifestations. The after care was left to the surgeons and the man recovered).

